# Honors to a Buffalo Physician

**Published:** 1885-01

**Authors:** 


### Honors to a Buffalo Physician.

Dr. Edward N. Brush, formerly of this city and lately First Assistant Physician at the New York State Lunatic Asylum at Utica, has recently been appointed to the charge of the Male Department of the Pennsylvania Hospital for the Insane at Philadelphia. We understand that this appointment was made at the suggestion of Dr. J. B. Chapin, superintendent, by the unanimous vote of the managers.

Dr. Brush is a native of this city, where he graduated in medicine and engaged in practice for some years thereafter. He was formerly one of the visiting physicians to the Buffalo General Hospital, one of the editors of this Journal and a lecturer in the Buffalo Medical College. He was appointed one of the physicians of the Lunatic Asylum at Utica in 1878, where he has since resided. He is a physician of varied and rare accomplishments, and he will carry to his new field a large experience in the care and management of the insane, which cannot fail to make his services of unusual value to both the State and its unfortunate wards.

We extend our hearty congratulations to the managers upon their rare good fortune in filling such a responsible position with so competent an officer; two conditions so very desirable to attain, yet, often, so difficult to fulfill.

---

Dr. Hughes Bennett, of London, has recently made a remarkable diagnosis, with localization of a brain tumor, and directed a surgical operation which led to its successful removal. It is believed to be the first of the kind thus treated. He diagnosed an encephalic morbid growth of limited size in the upper part of the fissure of Bolando, and requested a surgeon to trephine the skull over the suspected region. This was done by Mr. Rickman Godlee, and a mass of glioma, the size of a walnut, was extracted from under the gray matter of the upper part of the ascending frontal convolution. The operation was performed November 25th, and the patient was doing well on December 6th (*London Medical Times*). The chief symptoms which led Dr. Hughes to diagnose the extent and locality of the tumor were paroxysmal twitchings of the left side of the face, alternating with twitchings of the arm on the same side, followed by slowly progressive paralysis of the hand, and, later on, by twitchings of the eyelids and leg without paralysis. These symptoms were accompanied by double optic neuritis and violent headache.

---

Messrs. Mayer & Mammen, surgical and orthopædic instrument makers, have established themselves at No. 6 East Huron street, near Main street. These gentlemen are skilled workmen, direct from Tiemann's, of New York City. They repair and make all kinds of instruments, even the most delicate cutting eye instruments, and, to our personal knowledge, their work is first-class. They merit the support of the profession in this and surrounding vicinity.

